# Prevalence of smoking among secondary school male students in Jeddah, Saudi Arabia: a survey study

**DOI:** 10.1186/1471-2458-13-1010

**Published:** 2013-10-25

**Authors:** Hashim R Fida, Ismail Abdelmoneim

**Affiliations:** 1 Family and Community Medicine Department, King Abdulaziz University, Jeddah, Saudi Arabia

**Keywords:** Smoking, Prevalence, Male students, Secondary schools, Saudi Arabia

## Abstract

**Background:**

This study was conducted to examine the prevalence of smoking and the smoking habits among male secondary school students in Jeddah, Kingdom of Saudi Arabia and to assess their knowledge and attitudes towards smoking.

**Methods:**

A cross-sectional study was conducted in Jeddah, using a two-stage cluster sample that randomly selected four schools from 85 public secondary schools for males. Data were obtained through a self-administered questionnaire containing questions on personal background, smoking behavior, knowledge, and behavior and attitudes towards smoking. A total of 695 students responded to the questionnaires with an 87.4% response rate.

**Results:**

The age range of this student sample was 16–22 years. Two hundred fifty-eight (37%) of the study group were current smokers. The most common reasons given for smoking were personal choice (50.8%) and the peer pressure from smoker friends (32.8%). Many students researched the smoking hazards (68.1%), but only 47.6% knew about the bad effects of passive smoking. Two thirds of the smoking students wanted to quit smoking (63.2%), especially if suitable help was available, and 75.1% tried to quit. A third of the smoking students (36.8%) found it difficult to stop smoking in no-smoking areas.

**Conclusion:**

A well-planned integrated antismoking campaign is urgently required, especially among students and teachers. Our study revealed that smoking prevalence was high, which will lead to future high smoking-related health problems if proper preventive measures are not taken accordingly.

## Background

Despite its decline in developed countries, high rates of smoking are still found in developing countries [1]. Many developing countries have adopted preventive campaigns to combat smoking with varying rates of success [2]. Among the factors that need to be considered in a well-designed preventive program are the prevailing social factors and determinants that strengthen and perpetuate this habit in the specific social environment [3].

The sociodemographic factors related to smoking behavior have been studied extensively in those countries that succeeded in combating smoking [4,5]. However, in developing countries, work is required to define those factors and the various impacts and interactions with global influences in those societies from the overflow of information and advertisements from the relentless tobacco industry. This interaction is continuous and while the social determinants of smoking change over time as a result of societal pressures, the tobacco industry has evolved new methods to target potential customers [6].

Youth are the main target for these companies because they ensure the perpetuation of smoking in developing countries. Starting smoking before the age of 18 years is a factor for life-long smoking and for those smokers, quitting will be more difficult [7,8], which increases the burden of chronic illnesses later in life [9]. On the other hand, adolescence is a developmental period where behavior is influenced by accelerated changes affecting biological, emotional, cognitive, and social functions.

It is interesting to note how a conservative society such as Saudi Arabia, where smoking was socially, traditionally, and above all religiously banned, has participated in the tobacco smoking pandemic to achieve unprecedented levels of smoking prevalence [10]. Nowadays, smokers are thought to comprise about half of the population and Saudi Arabia is ranked fourth in worldwide cigarette imports with an annual increase in tobacco consumption at around 3%. Meanwhile, there are no regulations to prevent Saudi youths from purchasing or using tobacco by any means at the present relatively low cost [11].

The aim of this study was to estimate the prevalence rate of smoking among male students (aged 16–18 years) and to determine the potential factors and behaviors related to smoking in this age group. To our knowledge, there were no previous studies addressing this issue in male secondary schools in Jeddah, Saudi Arabia.

## Methods

Jeddah city is an important center for various activities in Saudi Arabia. A gateway city for more than 3 million pilgrims who pass through to Mecca every year, Jeddah is considered the most liberal city of the kingdom with a cosmopolitan population of 3.2 million inhabitants. Jeddah is responsible for a large proportion of the economic activities of Saudi Arabia, and more than a hundred international agencies are based here [12,13].

Our study involved male secondary school students. The initial sample size of this study was calculated to be around 400 students, but the investigators decided to double that number to account for an expected number of nonresponses and to omit incomplete questionnaires. The calculation was based on the assumption of a margin of error at 5%, a confidence interval of 95% and a response level of 50%. The total number of male students in Jeddah governmental secondary schools was 85000 and the sample included 11 schools in the north, seven schools in the middle, and 20 schools in the southern regions. The sampling method was a two-stage cluster method that randomly included governmental secondary schools from the three main regions of Jeddah for the first stage (north, one school; middle, one school; south, two schools). In the second stage, 25 full classes (average 30 students per class) from the three levels of selected secondary schools were chosen randomly by proportional allocation (10 classes from the first year, eight classes from the second year, and seven classes from the third year).

After completing these administrative steps, the study protocol and the required consent forms were verified and approved by the local ethical committee of the Faculty of Medicine at King Abdulaziz University. The self-administered questionnaire was succinct to encourage the students to respond. It comprised 29 questions that were modified and translated from previously used surveys in Saudi Arabia [14] including 13 questions about the socioeconomic profile of the respondents as well as smoking knowledge, practices, and attitudes (16 questions). A smoker in this study was considered one who ever smoked and continues to smoke, even irregularly, but who does not describe himself as a past smoker.

A team of four assistants was trained to ensure a unified method of data collection procedures. A preliminary pilot study was undertaken for 22 students and some questions were subsequently rephrased and modified accordingly. The data were collected on the first day of the school week (to reduce the chance of absenteeism) and during the lunch break in the same classrooms without the presence of any teacher or supervisor except the data collector. The session started by explaining the procedure and the questionnaires were subsequently distributed to the students. After about 15 minutes, the questionnaires were collected and no student was forced to return their questionnaire if they felt unwilling to return it.

The collected data were validated and processed using SPSS software (version XX;IBM SPSS,Armonk,NY). A bivariate analysis was performed with smoking as the dependent variable and sociodemographic factors as independent variables. The resulting significant variables were introduced into a logistic regression model to test the strength and level of significance of these variables as predictors of smoking. A descriptive analysis was performed to depict the behavioral factors associated with smoking. The level of significance was *P* < 0.05.

## Results

Eight hundred students were selected for this study; the response rate was about 87% (695 students).

The prevalence rate of smokers among students was 37.1%. Their age range was 16–22 years, with a mean age of 17.06 ± 0.80 years with no significant difference between ages of smokers (17.13 ± 0.82 years) and nonsmokers (17.01 ± 0.79 years). The mean duration of smoking among smokers was 33.3 ± 27.5 months.

Table 1 demonstrates the results of the bivariate analysis for the sociodemographic characteristics associated with smoking in adolescent male students. The parents’ education level is a significant factor associated with their children’s smoking habits. About 42% of students with a father with a secondary education or less were smokers compared with 34% of students who have a university-educated father (odds ratio (OR) = 1.42; 95% confidence interval (CI): 1.02–1.99). Similarly, about 40% of students with a less-than-university-level-educated mother were smokers compared with 31.3% for students with a university-educated mother (OR= 1.51; 95% CI: 1.07–2.43). Moreover, a student who has a smoker friend is significantly more liable to become a smoker (42.5%) than a student with nonsmoker friends (12.1%) (OR= 5.31; 95% CI: 2.88–9.94). The same pattern was found among smoker students when one or more persons at home were smokers (65.9%) compared with 34.4% of smoker students who came from smokefree homes (OR= 3.10; 95% CI: 2.21–4.35). Table 1 shows that the types of work performed by the parents, the household’s monthly income, and their housing conditions showed no statistically significant associations.

**Table 1:** Social factors and smoking among adolescent male students in Jeddah City, Saudi Arabia

**Factors**	**Smokers**	***P *** **value**	**OR (95% CI)**
Father’s education (n = 653)			
- High school or less	110 (42.0%)	<0.05	1.42 (1.02–1.99)
- University	132 (33.8%)		
Mother’s education (n = 634)			
- High school or less	145 (40.7%)	<0.05	1.51 (1.07–2.43)
- University	87 (31.3%)		
Father’s work (n = 695)			
- Professional	47 (32.6%)	0.21	0.78 (0.53–1.51)
- Nonprofessional	211 (38.3%)		
Mother’s work (n = 590)			
- Housewife	164 (37.9%)	0.44	1.16 (0.79–1.70)
- Working	54 (34.4%)		
Monthly income (n = 641)			
- <5000 SR	38 (40%)	0.70	1.09 (0.70–1.70)
- >5000 SR	207 (37.9%)		
House (n = 656)			
- Owned	168 (38.5%)	0.32	0.10 (0.71–1.40)
- Rented	85 (38.6%)		
Overcrowding index (n = 632)			
- >1 person/room	34 (66.6%)	0.95	0.98 (0.62–1.65)
- <1 person/room	199 (36.9%)		
Smoker friends (n = 695)			
- Yes	244 (42.5%)	<0.01	5.31 (2.88–9.94)
- No	14 (12.1%)		
Smokers at home (n = 669)			
- Yes	168 (65.9%)	<0.01	3.10 (2.21–4.35)
- No	87 (34.4%)		

OR, odds ratio.

95% CI: 95% confidence interval.

Table 2 shows the result of the logistic regression model involving the significant factors revealed by the bivariate analysis (Table 1). The results indicate that the significant factors in this model were reduced to only the presence of smoker friends and other smokers at home (OR = 4.81; 95% CI: 2.53–9.16 and OR= 2.85; 95% CI: 2.00–4.05, respectively).

**Table 2:** Result of the logistic regression model showing the impact of independent factors on smoking status as the dependent factor

**Factors**	**Significance**	**OR**	**95% CI**	
			**Lower**	**Upper**
Father’s education	0.852	1.037	0.707	1.520
Mother’s education	0.076	0.710	0.486	1.037
Smoker friends	0.000	4.812	2.528	9.156
Smokers in household	0.000	2.848	2.001	4.054
Constant	0.000	0.029		

95% CI: 95% confidence interval; OR: odds ratio.

Table 3 describes the behavioral factors related to smoking in adolescent male smoker students in this study. It shows that the prevalence of smoking is about 37%. Of these smoker students, 39.7% were mainly cigarette smokers, while 33.1% were nargile or shisha smokers and a substantial percentage of students (27.2%) combined different smoking methods. One third of students (35.7%) were regularly smoking one to five times/day. The highest percentage (50.8%) claimed smoking was their own personal choice and was not due to peer pressure, and thought that the majority (62.9%) of youth smoke for entertainment and not to imitate anyone. Most (63.2%) intend to quit or have already tried to quit (75.1%), especially if they were suitably helped (77.5%). Most (63.2%) did not feel uncomfortable in a nonsmoking area. Although many of them sought information about smoking risks, 52.4% did not know what passive smoking meant.

**Table 3:** Behavioral factors related to smoking in adolescent male students in Jeddah City, Saudi Arabia

**Factors**	**N**	**%**
Prevalence of smokers (n = 695)		
- Yes	258	37.1%
Type of smoking (n = 257)		
- Cigarettes	102	39.7%
- Nargile or shisha	85	33.1%
- A combination	70	27.2%
Smoking daily (n = 258)		
- Occasional	70	27.1%
- 1–5 times per day	92	35.7%
- 5–10 times per day	46	17.8%
- More than 10 times per day	50	19.4%
Why do you smoke? (n = 201)		
- Because my friends smoke	66	32.8%
- Because my parents smoke	33	16.4%
- Personal choice	102	50.8%
Why do other youth choose to smoke? (n = 205)		
- For entertainment	129	62.9%
- To show their virility	48	23.4%
- Imitation of others	28	13.7
Do you feel uncomfortable in non-smoking areas: (n = 234)		
- Yes	86	36.8%
Do you intent to quit? (n = 258)		
- Yes	163	63.2%
- No	53	20.5%
- Undecided	42	16.3%
Have you tried to quit before? (n = 209)		
- Yes	157	75.1%
Will you quit smoking if suitably helped? (n = 231)		
- Yes	179	77.5%
Did you research information about smoking risks? (n = 251)		
- Yes	171	68.1%
What are the effects of passive smoking? (n = 246)		
- Bad effect on others	117	47.6%
- No effect on smokers	18	7.3%
- Don’t know	111	45.1%

## Discussion

The prevalence of smoking among this group of male teenagers in Jeddah secondary schools was higher (37.1%) than the rate in some previous Saudi studies [14]. The prevalence cited by Abdalla *et al*. [15] was 34% among current male cigarette smokers (students who had smoked on one or more days in the 30 days preceding the survey), and 11.1% among males who were daily smokers. Similarly Al Ghobain *et al*. [16] found a smoking prevalence of 31.2% among male students aged 16–18 years in Riyadh and the significant smoking-related factors were having smoker friends and parents. One of the possible reasons for this finding is that our study included all who answered ‘yes’ to the question ‘are you a smoker?’ even if irregular, as long as they had not quit. In our opinion, smoking at that age is probably in the trial phase and irregular in a considerable number of youths. Nevertheless, this factor should be considered from a preventive perspective because smoking at that age is expected to become a lifelong habit in a proportion of students, which will make quitting more difficult [17].

The significant effect of the parents’ level of education on their children’s behavior is to be expected, especially in the Saudi society where children are of an age presumably still influenced by their home values and beliefs. Moreover, in most cases, a better-educated parent could deal more effectively and rationally with their children’s behavior [18,19].

The results of the logistic regression model showed that the factors of smoking peers and the role played by a smoking household member were the only significant variables in this model. At this age, peer pressure is expected to play an important role in the students’ behavior, especially when combined with the detrimental effect of the presence of a smoking household member [20]. This negative combination will probably result in a tendency towards being a smoker.

The smoking behavior of this age group reflects their eagerness to discover different types of smoking as shown by a mostly equal prevalence of different methods of smoking tobacco as cigarettes, shisha, nargile, and even combinations of methods. This behavior is considered a warning sign because more detrimental and strongly addictive narcotic substances have entered Saudi Arabia recently and are readily available to this young age group. However, in this study, family income had no effect on smoking prevalence, which indicates that the cost of regular methods of smoking is affordable compared with the expected prices of the more dangerous narcotic substances.

When asked why they or other youths smoke, most students denied that they started smoking because of peer pressure or relatives, but stated they smoked as a personal choice and for entertainment. Smoking as entertainment was reported in some studies where youths combined smoking with other entertainment [21]. An important point of interest concerning the personal choice to smoke is that students claim they will respect that personal choice if made by members of their own future families.

Among the positive aspects of this study was the finding that most students did not feel any discomfort from being prevented from smoking while in a nonsmoking zone, which probably indicated that the majority were still in a nonaddicted phase. Again, the majority of students intend to quit, especially if suitably supported, or had already tried to quit. Another positive finding is that most sought information about smoking risks. However, the accessed information was unclear since most did not know what passive smoking was. Such misinformation may cause health risks and negative repercussions for their peers and relatives, which calls for specifically tailored educational messages for this age group.

The limitations of this study were the exclusion of nongovernmental schools for the sake of a homogeneous sociodemographic student sample and the selection of only male students to overcome some administrative and social difficulties related to including female students in this study.

## Conclusions

This study illustrates the importance of the parental level of education on the smoking behavior of children, which is also influenced by the presence of a smoking member of the household. At the age when adolescents start to make social networks with their peers, the smoking behavior of peers has a considerable effect. Therefore, we recommend targeting parents through their children’s schools with suitable antismoking education programs in addition to programs targeting the students themselves.

## Competing interests

Financial competing interests

In the past five years, the authors did not receive reimbursements, fees, funding, or salaries from any organization that may in any way gain or lose financially from the publication of this manuscript, now or in future. The authors do not hold any stocks or shares in an organization that may in any way gain or lose financially from the publication of this manuscript, now or in future. The authors do not hold or currently apply for any patents relating to the content of the manuscript, nor have they received reimbursements, fees, funding, or salaries from an organization that holds or has applied for patents relating to the content of the manuscript. The authors declare that they do not have any other financial competing interests.

Nonfinancial competing interests

The authors do not have any nonfinancial competing interests (political, personal, religious, ideological, academic, intellectual, commercial, or any other) to declare in relation to this manuscript.

## Authors’ contributions

HF organized the field processes and the administrative work, designed the questionnaire, was responsible for data collection, cowrote the Introduction, and shared in data analysis. IA cowrote the Introduction, assisted in interpreting the results, wrote the Methods and Discussion, and performed statistical analysis. Both authors read and approved the final manuscript.

## Pre-publication history

The pre-publication history for this paper can be accessed here:

http://www.biomedcentral.com/1471-2458/13/1010/prepub
